# Effect of cranberry supplementation on liver enzymes and cardiometabolic risk factors in patients with NAFLD: a randomized clinical trial

**DOI:** 10.1186/s12906-021-03436-6

**Published:** 2021-11-19

**Authors:** Kourosh Masnadi Shirazi, Elham Shirinpour, Arman Masnadi Shirazi, Zeinab Nikniaz

**Affiliations:** 1 Liver and Gastrointestinal Diseases Research Center, Tabriz University of Medical Sciences, Tabriz, Iran; 2 Student research committee, Tabriz University of Medical Sciences, Tabriz, Iran

**Keywords:** Cranberry, NAFLD, Cardiometabolic, Liver enzymes

## Abstract

**Background:**

We aimed to evaluate the effect of cranberry supplementation on serum liver enzymes, hepatic steatosis, and cardiometabolic risk factors in patients with non-alcoholic fatty liver (NAFLD).

**Methods:**

In the present parallel-designed randomized controlled clinical trial, 110 patients with NAFLD were enrolled. The patients were randomized to receive 144 mg cranberry capsule or placebo for 6 months. The primary efficacy of the treatment was lipid profile, glycemic measurements, and liver enzyme levels.

**Results:**

The data were reported for 46 in the supplementation group and 48 in the placebo group. The patient’s mean (SD) age was 43.16 (11.08) years. No significant differences between groups were observed regarding the post-intervention level of liver enzyme. The mean after-intervention levels of total cholesterol (*p* < 0.001) and triglyceride (*p* = 0.01) were significantly lower in the intervention group compared with the placebo group. At the end of the study, the mean insulin and HOMA-IR levels were significantly lower in the cranberry group compared with the placebo group. Significantly more patients in the cranberry group experienced a decrease in steatosis level compared with the control group.

**Conclusion:**

The results of the present study showed that cranberry supplementation had a positive effect on some lipid profiles, insulin resistance, and hepatic steatosis in patients with NAFLD.

**Trial registration:**

IRCT20200725048200N1; first registration date: 11.8.2020.

**Supplementary Information:**

The online version contains supplementary material available at 10.1186/s12906-021-03436-6.

## Background

Nonalcoholic fatty liver disease (NAFLD) is defined by excessive accumulation of lipids in the liver, which is not induced by alcohol intake, drug use, or viral hepatitis [1]. The NAFLD prevalence was reported as 25.24% worldwide, with a high prevalence in the Middle East countries [2]. NAFLD is related to other diseases such as kidney and cardiovascular diseases representing the effects of this disease on the body [3].

Considering the burden of NAFLD, researchers have mainly focused on examining new and effective methods for the prevention and treatment of this disease [4]. Different disease management options such as lifestyle interventions, drug and vitamin supplements therapy, phlebotomy, and surgical interventions were suggested to accomplish on NAFLD patients [5]. However, the majority of these procedures are ineffective and some methods like various types of surgeries are invasive and can be associated with other complications. Therefore, better methods and medication had to be investigated for NAFLD treatment.

Nowadays, an increasing number of studies have focused on the efficacy of herbal medicine in NAFLD patients [6]. Some studies showed the positive effect of herbal medication along with lifestyle modification in patients with NAFLD [6]. Cranberry (*Vaccinium macrocarpon*) is a fruit with high content of different polyphenols [7]. Different animal studies showed the promising effect of cranberry on liver enzymes and hepatic steatosis [8, 9]. Moreover, various human studies have also focused on the effect of Cranberry capsules (240–1500 mg/day) [9–12] and Cranberry juice (240–750 ml/days) [13–19] on cardiometabolic risk factors and providing mixed results. Some studies reported the protective effect of cranberry on TC [11], LDL-C [11], HDL-C [19], FPG [16], and blood pressure [15]. However, the results of a recent systematic review and meta-analysis study concluded that cranberry supplementation has significantly positive effects on blood pressure and weight loss in patients with diabetes, and metabolic syndrome. However, no favorable effect was observed on glycemic measurements and lipid profile [20]. To the best of our knowledge, so far, only one study has assessed the effect of cranberry in patients with NAFLD [21]. Hormoznejad et al. have assessed the effect of 288 mg of cranberry supplementation for 3 months on cardiometabolic risk factors and steatosis grade in patients with NAFLD and showed a significantly greater reduction of alanine aminotransferase (ALT) and insulin in the cranberry group than in the placebo group. The intervention duration in this study was limited and they recommended long-term clinical trials in NAFLD patients [21].

Considering the high antioxidant capacity of cranberry and owing to the involvement of oxidative stress in the pathogenesis of NAFLD, we postulated that a hypocaloric diet along with cranberry supplementation maybe effective in the management of NAFLD. Due to the lack of studies in this regard, in the present clinical trial, we evaluated the effect of cranberry supplementation on serum liver enzymes, hepatic steatosis, and glycemic and lipid profiles in patients with NAFLD.

## Methods

### Patients

In the present parallel-designed triple-blind randomized controlled clinical trial, the previously diagnosed patients with NAFLD who were referred to the liver disease clinic of Imam Reza educational hospital, Tabriz, Iran from august 2020 were enrolled. The patients were diagnosed based on liver ultrasonography previously by expert gastroenterologists. Adult patients aged more than 18 years were included. The pregnant and breastfeeding patients, the patients with diabetes, other liver diseases, heart, renal and pulmonary failure, patients with alcohol intake, and the ones who used antioxidant and vitamin supplements other than vitamin E were ineligible.

One hundred and ten patients with NAFLD participated in the present trial. Simple randomization was done using a computer-generated randomization chart by the GraphPad QuickCalcs tool. A sequentially numbered sealed envelope was used to randomize participants into two groups. A researcher who did not play any role in the other part of the investigation developed the randomization chart and assigned the patients into the intervention groups. All participants who have received a hypocaloric diet of 500 kcal less per day than estimated energy requirements and vitamin E supplement. The patients in the intervention group (*n* = 55) received a single capsule of cranberry (144 mg) and the patients in the placebo group (*n* = 55) received the placebo for 6 months. The patients were advised to take the capsules after lunch. To increase the patients’ compliance to intervention, participants were fully informed regarding the trial before the initiation of interventions. Moreover, the pre-planned telephone call was undertaken to answer questions and address any issues that arise.

Cranberry, and placebo were purchased from Shari Company, Iran. The cranberry capsule includes 144 mg *Vaccinium macrocarpon* (equal to 13 g dried cranberry fruit). The placebo includes the same base formula without the active ingredient. Cranberry and placebo were the same, labeled as A and B, and ordered by a researcher who was not involved in other parts of the clinical trial. The patients, the outcome assessor, and the statistician were blind to group assignment.

All participants have signed full written consent. The ethics committee of Tabriz University of Medical Sciences approved the study protocol (Ethics code: IR.TBZMED.REC.1399.090). The study was carried out in accordance with the ethical guidelines of Tabriz University of medical sciences. The trial was registered at the Iranian registry of clinical trials (Identifier NO. IRCT20200725048200N1; first registration date: 11.8.2020).

The sample size was calculated using G-power software based on the result of a previous study [15] about the effect of cranberry juice on the glycemic indices and by the presumption of a two-sided significance level of 5% and power of 80% with equal allocation to the two arms that necessitate a sample size of 37 in each group. To allow for dropouts, 55 patients were recruited.

### Measurements

The participants were visited every month during the intervention. In all visits, compliance with the intervention and lifestyle modifications were checked. If participants consume > 80% of their prescribed medication were considered compliant.

### Evaluation of the therapeutic efficacy

The primary endpoints were lipid profile [total cholesterol (TC), triglyceride (TG), high-density lipoprotein cholesterol (HDL-C), and low-density lipoprotein cholesterol (LDL-C)], glycemic measurements [fasting blood sugar (FBS), and insulin level], liver enzymes [alanine aminotransferase (ALT), aspartate aminotransferase (AST), alkaline phosphatase (ALP)] levels.

Anthropometric characteristics, including weight, and height were measured at the beginning and end of the study. Height was measured to the nearest 0.1 cm using a tape measure. Weight was measured using a Seca weighing scale to the nearest 0.1 kg. BMI was also calculated as weight in kilograms (kg)/height in meters squared (m^2^).

After 10-h fasting, a blood sample was obtained. All measurements were done in the same laboratory and using the same procedures. The colorimetric method (Parsazmoun, Tehran, Iran) was used for measuring liver enzymes, FBS, TG, TC, and HDL-C levels. ELISA method (Monobind, USA) was used for measuring serum insulin level. The concentration of LDL-C was calculated using the Friedewald formula and the homeostatic model assessment insulin resistance index (HOMA-IR) was calculated according to Gayoso-Diz et al. formula: HOMA-IR = fasting glucose (mmol/l)* fasting insulin (lU/mL)/ 22.5.

### Statistical analysis

SPSS version 16 was used for statistical analysis. Kolmogorov-smirnov test was used for assessing data distribution. Mean and standard deviations (SD) were used for reporting the continuous variables and frequency and percentage were used for reporting categorical data. A paired sample t-test was used for comparison of the before- and after-intervention values in each group. For between-group comparisons, the chi-square test and independent t-test were used where appropriate. For comparison of the post-intervention values adjusted for age, sex, BMI, and baseline values, a one-way analysis of covariance (ANCOVA) was used. A *p*-value of less than 0.05 was considered significant.

## Results

**Fig. 1:**
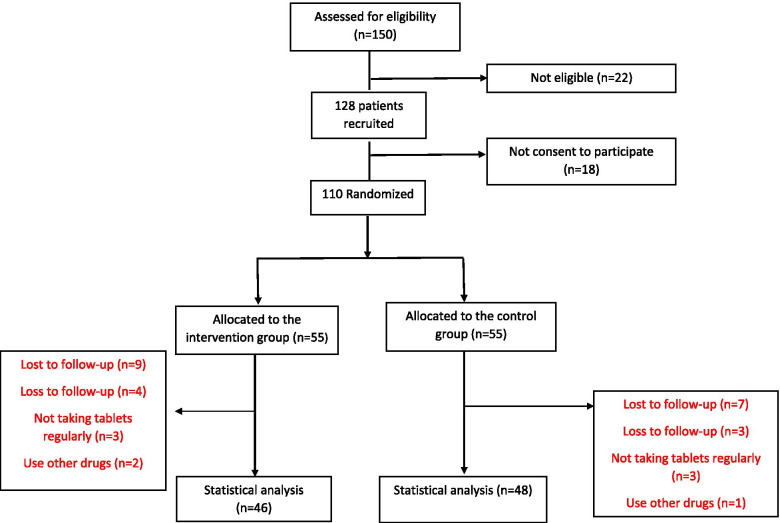
Flow chart of patients’ recruitment and analysis

From a total of 110 patients with NAFLD, nine patients in the intervention group and seven patients in the control group were lost to follow-up. The data were stated for 94 patients (46 in the cranberry group and 48 in the placebo group) (Fig. 1).

The mean (SD) age of the participants was 43.16 (11.08) years, 47.9% of them were male, and 76.6% of them were overweight or obese. The baseline demographic and clinical characteristics were not significantly different between the two groups (Table 1).

**Table 1:** the baseline characteristics of participants

Variables	Cranberry group (***n*** = 46)	Placebo group (***n*** = 48)	***p***-value *
**Age (years)**	43.20 ± 11.30	43.13 ± 10.98	0.97
**Sex n (%) male/female**	22 (47.8)/ 24 (52.2)	23 (47.9)/ 25 (52.1)	0.99
**BMI (kg/m**^**2**^**)**	28.17 ± 4.99	28.63 ± 4.15	0.63
**SBP (mmHg)**	127.39 ± 24.61	124.85 ± 23.20	0.60
**DBP (mmHg)**	79.35 ± 10.19	77.90 ± 9.05	0.46
**Total cholesterol (mg/dL)**	209.8 ± 64.13	205.60 ± 50.16	0.72
**LDL-C (mg/dL)**	144.13 ± 20.62	143.27 ± 20.02	0.83
**HDL-C (mg/dL)**	33.67 ± 4.21	34.46 ± 5.00	0.41
**TG (mg/dL)**	198.9 ± 64.99	181.50 ± 57.79	0.17
**AST (**IU**/L)**	37.22 ± 13.51	41.17 ± 16.69	0.21
**ALT (**IU**/L)**	42.74 ± 15.04	47.48 ± 18.35	0.17
**ALP (**IU**/L)**	251.57 ± 32.89	248.08 ± 27.18	0.57
**FBS (mg/dL)**	105.22 ± 9.04	105.81 ± 9.08	0.75
**Insulin (μ/mL)**	10.38 ± 3.09	10.65 ± 3.02	0.67
**HOMA-IR**	2.78 ± 0.99	2.84 ± 0.98	0.74
**Steatosis grade n (%)**			
**Grade 1**	13 (28.3)	16 (33.3)	0.8**
**Grade 2**	28 (60.9)	26 (54.2)	
**Grade 3**	5 (10.9)	6 (12.5)	

*p-value of independent t-test **p-value of chi-square test

*SBP* systolic blood pressure, *DBP* diastolic blood pressure, *LDL-C* low-density lipoprotein-cholesterol, *HDL-C* High-density lipoprotein-cholesterol, *TG* triglyceride, *AST* alanine aminotransferase, *AST* aspartate aminotransferase, *ALP* alkaline phosphatase, *FBS* fasting blood sugar, *HOMA-IR* Homeostatic model assessment-insulin resistance

As shown in Table 2, the mean liver enzyme levels were significantly decreased in the placebo group (*p* < 0.001), however, in the intervention group, there was a significant reduction in only the ALP levels. The results of the ANCOVA test showed that there were no significant differences between groups regarding the after-intervention level of liver enzymes after adjusting for age, sex, BMI, and baseline values.

**Table 2:** comparison of the mean anthropometric, glycemic measurement, lipid profile, and liver enzymes before and after study between two groups

Variable	Cranberry group (***n*** = 46)		***p***-value*	Placebo group (***n*** = 48)		***p***-value*	***p***-value**
	Before	After		Before	After		
**BMI (kg/m**^**2**^**)**	28.17 ± 4.99	28.22 ± 5.06	0.32	28.63 ± 4.15	28.42 ± 4.07	0.68	0.34
**SBP**	127.39 ± 24.61	126.85 ± 25.01	0.22	124.85 ± 23.20	124.79 ± 24.25	0.89	0.37
**DBP**	79.35 ± 10.19	79.78 ± 10.21	0.25	77.90 ± 9.05	78.65 ± 9.32	0.07	0.62
**Liver enzymes**							
**ALT**	42.74 ± 15.04	39.54 ± 16.95	0.18	47.48 ± 18.35	38.69 ± 14.20	< 0.001	0.27
**AST**	37.22 ± 13.51	32.98 ± 14.33	0.05	41.17 ± 16.69	31.98 ± 12.48	< 0.001	0.27
**ALP**	251.57 ± 32.89	224.33 ± 54.12	0.01	248.08 ± 27.18	221.40 ± 47.19	< 0.001	0.77
**Lipid profile measurements**							
**Total cholesterol**	209.8 ± 64.13	189.02 ± 63.62	< 0.001	205.60 ± 50.16	200.29 ± 52.14	0.002	< 0.001
**LDL-C**	144.13 ± 20.62	133.96 ± 20.71	< 0.001	143.27 ± 20.02	135.17 ± 19.34	0.24	0.23
**HDL-C**	33.67 ± 4.21	38.28 ± 4.84	< 0.001	34.46 ± 5.00	38.0 ± 4.99	< 0.001	0.09
**TG**	198.9 ± 64.99	190.54 ± 65.22	< 0.001	181.50 ± 57.79	188.54 ± 66.80	< 0.001	0.01
**Glycemic measurements**							
**FBS**	105.22 ± 9.04	98.65 ± 8.70	0.001	105.81 ± 9.08	100.48 ± 7.47	< 0.001	0.28
**Insulin**	10.38 ± 3.09	5.62 ± 2.04	< 0.001	10.65 ± 3.02	10.06 ± 2.94	< 0.001	< 0.001
**HOMA-IR**	2.78 ± 0.99	1.39 ± 0.62	< 0.001	2.84 ± 0.98	2.51 ± 0.85	< 0.001	< 0.001
**Steatosis grade n (%)**							
**No steatosis**	0	14 (30.4)	0.04#	0	0	< 0.001#	0.001#
**Grade 1**	13 (28.3)	18 (39.1)		16 (33.3)	24 (50)		
**Grade 2**	28 (60.9)	13 (28.3)		26 (54.2)	20 (41.7)		
**Grade 3**	5 (10.9)	1 (2.2)		6 (12.5)	4 (8.3)		

* Within-group comparisons, *P*-value of paired t-test

** Between-group comparisons; *p*-value of ANCOVA adjusted for age, sex, and baseline values

# *p*-value of chi-square test

*SBP* systolic blood pressure, *DBP* diastolic blood pressure, *LDL-C* low-density lipoprotein-cholesterol, *HDL-C* High-density lipoprotein-cholesterol, *TG* triglyceride, *AST* alanine aminotransferase, *AST* aspartate aminotransferase, *ALP* alkaline phosphatase, *FBS* fasting blood sugar, *HOMA-IR* Homeostatic model assessment-insulin resistance

In the term of lipid profile measurements, the mean after-intervention levels of total cholesterol (*p* < 0.001) and triglyceride (*p* = 0.01) were significantly lower in the intervention group compared with the placebo group after adjusting for baseline values, age, sex, BMI and baseline values (Table 2).

The mean level of glycemic indices was significantly decreased in both groups. However, the results of the ANCOVA test indicated that that at the end of the intervention, the mean insulin, and HOMA-IR levels were significantly lower in the cranberry group compared with the placebo group.

**Fig. 2:**
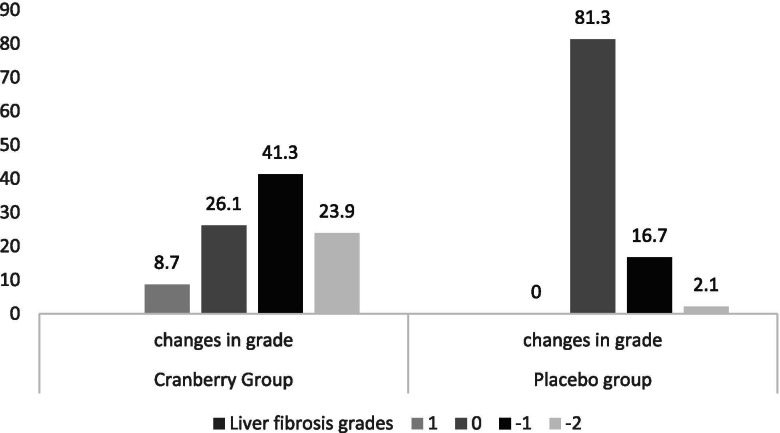
Changes in hepatic steatosis grades in two groups

The changes in hepatic steatosis grade are shown in Fig. 2. Significantly more patients in the cranberry group experienced a decrease in steatosis level compared with the control group (*P* < 0.01). Moreover, 8.7% of patients in the cranberry group experienced a one-point increase in steatosis level and the steatosis level did not increase in any patients in the control group, however, the differences were not statistically significant between groups (*p* = 0.06).

## Discussion

This RCT assessed the effect of cranberry supplementation on cardiometabolic risk factors and liver function tests in patients with NAFLD. The results showed that in the term of cardiometabolic risk factors, the mean reduction in total cholesterol, triglyceride, insulin, and HOMA-IR levels in the cranberry group was significantly higher compared with the placebo group. The effect of cranberry supplementation of glycemic measurements was in agreement with the result of a previous study in patients with NAFLD [21]. Hormoznejad et al. in patients with NAFLD showed that cranberry supplementation had a significant positive effect on insulin and HOMA-IR levels in patients with NAFLD [21]. In studies with patients with type 2 diabetes, the positive effect of cranberry supplementation on insulin levels, and HOMA-IR have been shown [22]. The favorable effect of cranberry on insulin sensitivity reported in this study cannot be related to variations in energy and, bodyweight since no changes were observed in these variables between the two groups. The positive effect of cranberry on glycemic measurement may be because of the improved insulin sensitivity [23].

The result of this study indicated that the mean serum FBS was significantly decreased in both groups. Although the mean decrease in serum FBS was higher in the cranberry group compared with the placebo group, the differences were not statistically significant. This finding is in line with the result of a previous study [21]. Some previous studies reported a significant decrease in serum glucose level by cranberry consumption [16, 24, 25]. The observed decrease in FBS in the cranberry group may partly because of the effect of cranberry on a delay in the gastric uptake of glucose or distribution of glucose to insulin-sensitive tissues [24]. Moreover, studies indicated that vitamin E and calorie restriction had a favorable effect on serum glucose levels [26, 27]. So, the significant decrease in serum FBS in the placebo group may be explained by the consumption of vitamin E and restriction of daily calories.

In the present study, we also observed significant differences in changes in TC and TG levels in the cranberry group compared with the placebo group. This finding is opposed to the result of a previous study in NAFLD patients [21]. This discrepancy may be partly related to differences in the intervention types between the two studies. The patients in the present study received vitamin E and energy restriction as routine treatments, however, in Hormoznejad et al. study the patients only received energy restriction as a routine treatment. A previous study on animal models showed the synergistic antioxidant effect of vitamin E and anthocyanins. Antioxidants in fruits had a lipid-lowering effect in a previous clinical trial [28]. Moreover, the duration, and supplement dosage were different in the two studies. In patients with type two diabetes, Lee et al. also showed a positive effect of cranberry supplementation on total cholesterol levels but not triglyceride levels [11]. A systematic review and meta-analysis study that reviewed the effect of cranberry supplementation on cardiometabolic risk factors also did not observe the significant effect of cranberry on lipid profile [20]. Our findings were inconsistent with the results of this meta-analysis that may be partly owing to the differences in including populations. We included the patients with NAFLD, however, none of the included studies in the meta-analysis involved the NAFLD patients and mostly studies the patients with type 2 diabetes or metabolic syndrome.

The observed positive effect of cranberry supplementation on lipid profile in the present study may be somewhat due to the polyphenol content of cranberry. Studies have indicated high content of tannins in cranberry increases the uptake of cholesterol in the liver [29]. Moreover, tannins may increase the excretion of cholesterol by binding to the bile acids in the intestine [30, 31].

We did not observe significant differences between the cranberry and placebo groups in terms of changes in liver enzyme levels. The only human study that assessed the effect of cranberry supplementation in patients with NAFLD reported significant differences between cranberry and placebo groups regarding changes in ALT level but not AST and ALP levels [21]. Faheem et al. also showed the effect of cranberry on decreasing liver enzymes level in rat models [8]. Other studies in fat models also indicated the promising effect of anthocyanins on liver enzyme levels [9]. A plausible explanation for the observed discrepancy between the results might be related to the differences in cranberry doses used in these studies. In the present study, we used the dose of 144 mg/day, however, Hormoznejad et al. used higher doses of cranberry (288 mg/day).

We observed significant differences between the two groups regarding changes in hepatic steatosis status. In an animal study, Anhe et al. also reported significant changes in steatosis status following cranberry supplementation [23]. Hormoznejad et al. also reported a significant reduction in hepatic steatosis in both cranberry and placebo groups [21]. The probable explanation for the positive effect of cranberry on hepatic steatosis may be related to the activation of PPAR-*α* by Pterostilbene, a stilbenoid found in cranberry. The activation of PPAR- *α* modulates pathways controlling the increased fatty acid *β*-oxidation and decreased triglyceride content in the liver and lowers plasma lipid levels in animal models [32]. Moreover, studies indicated that anthocyanins found in cranberry had a positive effect on liver steatosis in animal models [8, 9, 33, 34]. In the present study, the grade of steatosis was increased in four patients in the cranberry group. We postulated that other factors that were not controlled in the present study such as genetic variations, consumption of fructose, monounsaturated fatty acids, and trans-fatty acids may aggravate NAFLD, or alcohol use may responsible for this observation [35]. Although none of the patients in the present study reported the consumption of alcohol, however as alcohol consumption is illegal in Iran, most patients did not report the real amount of alcohol consumption.

The results of the present study should be interpreted considering the potential limitations of the study. We did not measure serum inflammatory and oxidative indices. Moreover, we assessed the steatosis status using the ultra-sonographic examination. Although histological assessment is the best way of assessing hepatic steatosis since liver biopsy is an invasive procedure we used ultrasound technique as an appropriate method for monitoring NAFLD [36].

Moreover, we did not control all confounders such as genetic polymorphism and nutrient intake that may affect the findings.

## Conclusion

In conclusion, the results of the present study showed that cranberry supplementation had a positive effect on some lipid profiles, insulin resistance, and hepatic steatosis in patients with NAFLD. However, considering the limitations of the study, more long-term studies with larger sample size and more valid methods of assessing hepatic steatosis are needed to confirm these preliminary results.

## Supplementary Information

**Additional file 1.**

## Data Availability

The datasets generated and/or analyzed during the current study are not publicly available due to due institution’s policy but are available from the corresponding author on reasonable request.

## Abbreviations

HOMA-IR: Homeostatic model assessment insulin resistance index
ALP: Alkaline phosphatase
AST: Aspartate aminotransferase
ALT: Alanine aminotransferase
FBS: Fasting blood sugar
LDL-C: Low-density lipoprotein cholesterol
HDL-C: High-density lipoprotein cholesterol
TG: Triglyceride
TC: Total cholesterol
NAFLD: Nonalcoholic fatty liver disease
