# Supplementary material for: Effect of cranberry supplementation on liver enzymes and cardiometabolic risk factors in patients with NAFLD: a randomized clinical trial
Source: BMC Complement Med Ther. 2021 Nov 19;21:283. doi: 10.1186/s12906-021-03436-6 (PMC8603491; doi:10.1186/s12906-021-03436-6)
Supplement: Supplementary file 1 — Additional file 1. [file 12906_2021_3436_MOESM1_ESM.docx]

**Project summary**

Considering the high antioxidant capacity of cranberry and owing to the involvement of oxidative stress in the pathogenesis of NAFLD, we postulated that lifestyle modification along with cranberry supplementation may be effective in the management of NAFLD. Due to the lack of studies in this regard, in the present clinical trial, we aimed to evaluate the effect of cranberry supplementation on serum liver enzymes, hepatic steatosis, and glycemic and lipid profiles in patients with NAFLD.

### General information

# Protocol title, protocol identifying number (if any), and date: Evaluation of the efficacy of Cranbery in management of non-alcoholic Fatty Liver Patients, Identifier NO. IRCT20200725048200N1; first registration date: 11.8.2020.

Name and address of the sponsor/funder: Liver and gastrointestinal diseases research center, Tabriz University of medical sciences, Tabriz, Iran

Name and title of the investigator(s) who is (are) responsible for conducting the research, and the address and telephone number(s) of the research site(s), including responsibilities of each: Dr Kourosh Masnadi Shirazi, Liver and gastrointestinal diseases research center, Tabriz University of medical sciences, Tabriz, Iran. TEL: 04133367473

**Rationale & background information**

Nonalcoholic fatty liver disease (NAFLD) is defined by excessive accumulation of lipids in the liver which is not induced by alcohol intake, drug use, or virus hepatitis [1]. The NAFLD prevalence was reported as 25.24% worldwide, with high prevalence in the Middle East countries [2]. NAFLD is related to other diseases such as kidney and cardiovascular diseases representing the effects of this disease on the body [3].

Considering the burden of NAFLD, researchers have mainly focused on examining new and effective methods for the prevention and treatment of this disease [4]. Different disease management options such as lifestyle interventions, drug and vitamin supplements therapy, phlebotomy, and surgical interventions were suggested to accomplish on NAFLD patients [5]. However, the majority of these procedures are not effective and some methods like various types of surgeries are invasive and can be associated with other complications. Therefore, better methods and medication were required to be investigated for NAFLD treatment.

Nowadays an increasing number of studies have focused on the efficacy of herbal medicine in NAFLD patients [6]. Some studies showed the positive effect of the medication along with lifestyle modification in patients with NAFLD [6]. Cranberry (Vaccinium macrocarpon) is a fruit with high content of different polyphenols [7]. Considering the high polyphenol content of cranberry, different studies have focused on its effect on cardiometabolic risk factors and providing mixed results. In a recent systematic review and meta-analysis study that reviewed 10 studies, it has been shown that cranberry supplementation has significantly positive effects on blood pressure and weight loss in patients with diabetes, and metabolic syndrome. However, no favorable effect was observed on glycemic measurements and lipid profile [8]. To the best of our knowledge, so far, only one study assessed the effect of cranberry in patients with NAFLD [9]. Hormoznejad et al have assessed the effect of 288 mg of cranberry supplementation for three months on cardiometabolic risk factors and steatosis grade in patients with NAFLD and showed a significantly greater reduction of alanine aminotransferase (ALT) and insulin in the cranberry group than in the placebo group. The intervention duration in this study was limited and they recommended long-term clinical trials in NAFLD patients [9].

Considering the high antioxidant capacity of cranberry and owing to the involvement of oxidative stress in the pathogenesis of NAFLD, we postulated that lifestyle modification along with cranberry supplementation may be effective in the management of NAFLD. Due to the lack of studies in this regard, in the present clinical trial, we aimed to evaluate the effect of cranberry supplementation on serum liver enzymes, hepatic steatosis, and glycemic and lipid profiles in patients with NAFLD.

**References (of literature cited in preceding sections)**

1. McCullough AJ: **The clinical features, diagnosis and natural history of nonalcoholic fatty liver disease**. *Clinics in liver disease* 2004, **8**(3):521-533.

2. Younossi ZM, Koenig AB, Abdelatif D, Fazel Y, Henry L, Wymer M: **Global epidemiology of nonalcoholic fatty liver disease—meta‐analytic assessment of prevalence, incidence, and outcomes**. *Hepatology* 2016, **64**(1):73-84.

3. Younossi Z, Anstee QM, Marietti M, Hardy T, Henry L, Eslam M, George J, Bugianesi E: **Global burden of NAFLD and NASH: trends, predictions, risk factors and prevention**. *Nature reviews Gastroenterology & hepatology* 2018, **15**(1):11-20.

4. Mitra S, De A, Chowdhury A: **Epidemiology of non-alcoholic and alcoholic fatty liver diseases**. *Translational gastroenterology and hepatology* 2020, **5**.

5. Beaton MD: **Current treatment options for nonalcoholic fatty liver disease and nonalcoholic steatohepatitis**. *Canadian Journal of Gastroenterology* 2012, **26**(6):353-357.

6. Xu Y, Guo W, Zhang C, Chen F, Tan HY, Li S, Wang N, Feng Y: **Herbal medicine in the treatment of non-alcoholic fatty liver diseases-efficacy, action mechanism, and clinical application**. *Frontiers in Pharmacology* 2020, **11**.

7. Narwojsz A, Tańska M, Mazur B, Borowska EJ: **Fruit physical features, phenolic compounds profile and inhibition activities of cranberry cultivars (Vaccinium macrocarpon) compared to wild-grown cranberry (Vaccinium oxycoccus)**. *Plant Foods for Human Nutrition* 2019, **74**(3):300-306.

8. Pourmasoumi M, Hadi A, Najafgholizadeh A, Joukar F, Mansour-Ghanaei F: **The effects of cranberry on cardiovascular metabolic risk factors: A systematic review and meta-analysis**. *Clinical Nutrition* 2020, **39**(3):774-788.

9. Hormoznejad R, Mohammad Shahi M, Rahim F, Helli B, Alavinejad P, Sharhani A: **Combined cranberry supplementation and weight loss diet in non-alcoholic fatty liver disease: a double-blind placebo-controlled randomized clinical trial**. *Int J Food Sci Nutr* 2020, **71**(8):991-1000.

**Study goals and objectives**

Determining the effect of cranberry extract on the response to treatment regimen of fatty liver patients on liver enzymes, ultrasound grade, lipid profile and serum glucose levels

**Study design**

double-blind parallel-designed randomized controlled clinical trial

**Methodology**

- In the present double-blind randomized controlled clinical trial, patients with NAFLD who were referred to the liver diseases clinic of Imam Reza educational hospital, Tabriz, Iran were enrolled. The patients were diagnosed based on liver ultrasonography previously by expert gastroenterologists. The adult patients aged more than 18 years were included. The pregnant and breastfeeding patients, the patients with diabetes, other liver diseases, heart, renal and pulmonary failure, patients with alcohol intake, and the ones who used antioxidant and vitamin supplements other than vitamin E were ineligible.
- One hundred and ten patients with NAFLD were randomized into two groups by a computer-generated randomization chart. All participants have received a hypocaloric diet of 500 kcal less per day than estimated energy requirements and vitamin E supplement. The patients in the intervention group (n=55) were received 144 mg cranberry capsule and the patients in the placebo group (n=55) were received the placebo for 6 months.
- Cranberry and placebo were purchased from Shari Company, Iran. The cranberry capsule includes 144 mg of Vaccinium macrocarpon (equal to 13 g dried cranberry fruit). The placebo includes the same base formula without the active ingredient. Cranberry and placebo were the same, labeled as A and B, and ordered by a researcher who was not involved in other parts of the clinical trial.
- All participants have signed the full written consent. The ethics committee of Tabriz University of medical sciences approved the study protocol (Ethics code: IR.TBZMED.REC.1399.090). The trial was registered at the Iranian registry of clinical trials (Identifier NO. IRCT20200725048200N1; first registration date: 11.8.2020).
- The sample size was calculated using g-power software based on the result of a previous study [10] about the effect of cranberry juice on the glycemic indices and by presumption of a two-sided significance level of 5% and power of 80% with equal allocation to the two arms that necessitate a sample size of 37 in each group. To allow for dropout, 55 patients were recruited.
- **Measurements**:
- The participants were visited every month during the intervention. In all visits, compliance with the intervention and also lifestyle modifications were checked. If participants consume >80% of their prescribed medication were considered compliant.
- **Evaluation of the therapeutic efficacy**
- The primary efficacy of the treatment was lipid profile [total cholesterol (TC), triglyceride (TG), high-density lipoprotein cholesterol (HDL-C), and low-density lipoprotein cholesterol (LDL-C)], glycemic measurements [fasting blood sugar (FBS), and insulin level], liver enzymes [alanine aminotransferase (ALT), aspartate aminotransferase (AST), alkaline phosphatase (ALP)] levels.
- Anthropometric characteristics, including weight, and height were measured at the beginning and the end of the study. Height was measured to the nearest 0.1 cm using a tape measure. Weight was measured using a Seca weighing scale to the nearest 0.1 Kg. BMI was also calculated as weight in kilograms (kg)/height in meters squared (m)2
- After 10-hour fasting, a blood sample was obtained. All measurements were done in the same laboratory and using the same procedures. The colorimetric method (Parsazmoun, Tehran, Iran) was used for measuring liver enzymes, FBS, TG, TC, and HDL-C levels. ELISA method (Monobind, USA) was used for measuring serum insulin level. The concentration of LDL-C was calculated using the Friedewald formula and the homeostatic model assessment insulin resistance index (HOMA-IR) was calculated according to Gayoso-Diz et al formula: HOMA-IR= Fasting glucose (mmol/l)* fasting insulin (lU/mL)/ 22.5

Assessed for eligibility (n=150)

Not eligible (n=22)

128 patients recruited partiipate

Not consent to participate (n=18)

110 Randomized

Allocated to the control group (n=55)

Allocated to the intervention group (n=55)

Lost to follow-up (n=9)

Lost to follow-up (n=7)

Died (n=1)

Statistical analysis (n=46)

Statistical analysis (n=48)

**Figure 1: Flow chart of patients’ recruitment and analysis**

**Safety considerations**

The adverse effects were checked weekly, and the patients asked to report any complication during study.

**Data management and statistical analysis**

SPSS version 16 was used for statistical analysis. Kolmogorov-Smirnov test was used for assessment of data distribution. Mean and standard deviations (SD) were used for reporting the continuous variables and the frequency and percent were used for reporting categorical data. A paired sample t-test was used for comparison of the before- and after-intervention values in each group. For between-group comparisons, the chi-square test and independent t-test were used where appropriate. For comparison of the post-intervention values adjusted for age, sex, and baseline values, one-way analysis of covariance (ANCOVA) was used. A p-value of less than 0.05 was considered significant.

**Ethics**

The ethics committee of Tabriz University of medical sciences approved the study protocol (Ethics code: IR.TBZMED.REC.1399.090).

**Informed consent forms**

All patients signed the informed consent form before participating in the study.

- **Research protocol: part 2**
- **Budget**
- Liver and gastrointestinal diseases research center, Tabriz University of medical sciences, Tabriz, Iran
